# Experiments Proving That Life May Be Preserved in Warm-Blooded Animals after the Destruction of a Considerable Part of the Spinal Marrow

**Published:** 1852-05

**Authors:** E. Brown-Séquard

**Affiliations:** Paris


*Experiments proving that life may be preserved in warm-blooded animals after the destruction of a considerable part of the spinal marrow.* By E. Brown-Séquard, M. D., of Paris.

It was believed by all Physiologists, until within the last three years, that the destruction of even a small portion of the spinal marrow was a cause of rapid death. Some experiments, the results of which I communicated to the Society of Biology, at Paris, on the 2d of December, 1848, induced me to believe that death, after destruction of a part of the spinal marrow, is more in consequence of hemorrhage than of any thing else. Since that time I have discovered that pigeons and many other birds are able to live and even to grow, after the destruction of nearly half of the length of the cord. (See the *Comptes Rendus et Mémoires de la Soc. de Biologie*, tom. 2, p. 28 and p. 49.) But I had never seen life continue more than ten days in mammals, after the destruction of the whole lumbar spinal cord.

Recently, in New York, from experiments performed upon a young cat, I have discovered that life may persist more than seventeen days after the destruction of that part of the spinal cord which extends from the eleventh or twelfth costal vertebra to the sacrum. The animal is still living. Although paraplegic, it appears to be in good health. Since the operation was performed, it has evidently grown much. In this case the animal was operated upon after having been rendered insensible by chloroform, a measure which I found useful in preventing a great loss of blood, and the general nervous disturbance consequent upon such an operation.

Although unique till now, this fact proves that the continuance of life is possible in one species of mammals, after the destruction of a considerable part of the spinal cord.

From what I have seen in this last experiment, as well as in the large number of experiments I have performed during the last three or four years, I think I am authorized in concluding that the well known opinions of Legallois, Wilson Philip, Krimer, Chossat and others, in relation to the influence of the spinal marrow upon the heart, the stomach and lungs, upon the urinary secretion, and upon animal heat, are quite erroneous.

